# Effect of epigallocatechin-3-gallate (EGCG) on cognitive functioning and the expression of APP and BDNF in the hippocampus of rats with streptozotocin -induced Alzheimer-like disease

**DOI:** 10.1016/j.bbrep.2025.101930

**Published:** 2025-01-31

**Authors:** Farnaz Ghayour Babaei, Ehsan Saburi, Fatemeh Forouzanfar, Mohadese Asgari, Zakieh Keshavarzi, Vahid Hajali

**Affiliations:** a Department of Biology, Ferdowsi University of Mashhad, Mashhad, Iran; b Medical Genetics and Molecular Medicine Department, Faculty of Medicine, Mashhad University of Medical Sciences, Mashhad, Iran; c Department of Neurosciences, Mashhad University of Medical Sciences, Mashhad, Iran; d Student Research Committee, Faculty of Medicine, Mashhad University of Medical Sciences, Mashhad, Iran; e Natural Product and Medicinal Plants Research Center, North Khorasan University of Medical Sciences, Bojnurd, Iran

**Keywords:** Alzheimer's disease, EGCG, Green tea, Spatial memory, Neurogenesis, BDNF

## Abstract

We aimed to investigate the potential therapeutic effects of the active substance of green tea, epigallocatechin-3-gallate (EGCG), on behavioral phenotypes and markers of neurogenesis in an Alzheimer disease (AD) rat model. The groups included sham, AD, and three AD groups receiving orally EGCG with different doses of 25, 50, and 100 mg/kg. The AD model was induced by intracerebroventricular (icv) injection of streptozocin (STZ) at a dose of 3 mg/kg. Spatial learning and memory were evaluated in the Morris water maze (MWM) test. Real-time PCR assay was used for evaluating the expression of beta-amyloid precursor protein (APP) and brain-derived neurotrophic factor (BDNF) in the hippocampus of animals. STZ disrupted the function of animals in MWM acquisition phase by almost 65 % and all doses of EGCG could return the learning parameters to those of control animals. STZ also impaired the memory function (P < 0.05) and a dose of 25 mg/kg EGCG could significantly return it to the control level (29 % vs 53 %, P < 0.01). Hippocampal APP gene expression was increased in the AD group and EGCG with dose 25 mg/kg decreased it significantly (P < 0.05). AD animals had decreased levels of hippocampal BDNF and treating with dose 25 mg/kg of EGCG could significantly increase it (P < 0.05). EGCG with dose 25 mg/kg can improve spatial memory deficits in AD model rats. It may be due to the impact on the expression of hippocampal factors involved in AD pathology. These findings could provide a beneficial insight for developing novel, safe, and efficient natural compounds for preventing or alleviation AD symptoms in humans.

## Highlights

• Spatial learning and memory was impaired in Alzheimer's disease (AD) animals.
• All administrated doses of EGCG restored learning in AD rats.
• Only dose 25 mg/kg of EGCG could restore memory deficits of AD rats to the control levels.
• Only dose 25 mg/kg of EGCG could restore the alteration of hippocampal APP and BDNF gene expression of AD rats.

## Graphical abstract

**Figure:**
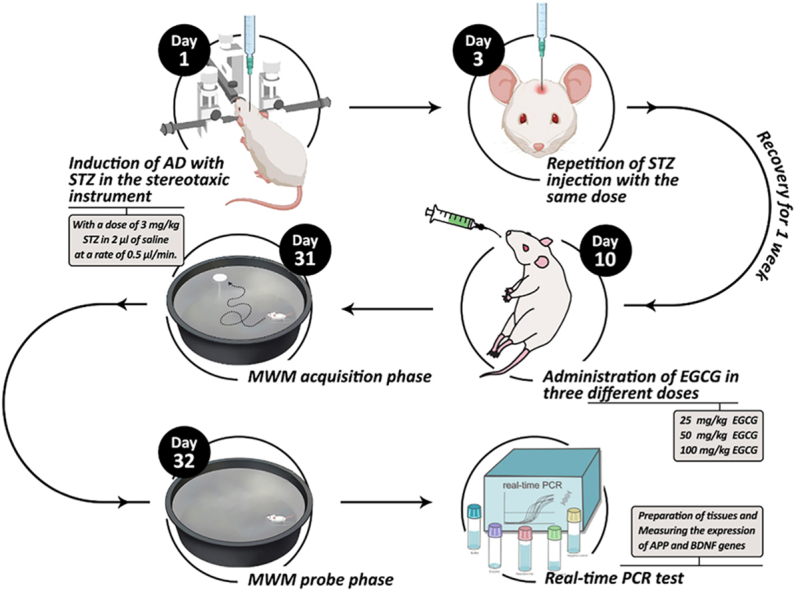

## 1 Introduction

Alzheimer's disease (AD) is a neurodegenerative condition linked to the accumulation of tau and amyloid deposits in the brain that results in behavioral abnormalities along with progressive cognitive and functional impairments. Around 50 million individuals worldwide suffer from dementia, and as the population ages, the number of patients is predicted to grow 3–4 times by 2050, raising the risk of disability, the burden of disease, and the healthcare costs. There has been an increase in research aimed at treating dementia because the condition is increasingly common in those over 60. The disease primarily affects those over 65, while 10 % of AD cases have been observed to have early-onset variations [1,2].

Patients who have AD in its most severe stage are totally unable to carry out their everyday tasks [3]. Due to the vague etiology and multifaceted pathogenesis, no definitive treatment or prevention strategy have been developed since AD was first recognized more than a century ego. Various drugs have passed clinical trials for different stages of AD. However, none have demonstrated a robust efficacy on enhancing general function or slowing cognitive deterioration [4].

Green tea (Camellia sinensis) has long been recognized for its potent antioxidant polyphenols, known as catechins, which have been linked to a lower risk of diabetes, Parkinson's, Alzheimer's, stroke, obesity, and cardiovascular illnesses [5]. It is currently a popular beverage around the world. Green tea's primary catechins are Epigallocatechin (EGC), Epicatechin-3-gallate (ECG), and Epigallocatechin-3-gallate (EGCG) [6].

One popular antioxidant molecule is EGCG. It is the primary catechin (50 %) found in green tea and its antioxidant, anti-inflammatory, anti-microbial, anti-cancer, anti-obesity, anti-aging, anti-amyloid and neuroprotective properties have been proven [7]. EGCG may also improve gap junction communication of the cells, which would shield cells from the formation of tumors [8,9].

No effective therapeutic strategy has yet been developed to treat AD or delay its progression, despite the high prevalence and financial burden in recent years. There has been a lot of interest in the use of plant-active chemicals in preventing or slowing the progression of neurodegenerative diseases such as AD [1]. Dietary antioxidants, particularly plant polyphenols, may help prevent or postpone the beginning of AD, according to epidemiological research [10]. A typical example is Mediterranean diet that known for its high absorption of fruits, vegetables, grains, and etc. It has been suggested that the greater adherence to the Mediterranean Diet my improve cognitive function, delay or prevent dementia and reduce the risk of AD [11].

Given the partial and limited efficacy of existing medications which has led to focus on developing more efficient and safe compounds for preventing or alleviation AD in recent years and also considering the discrepancies in the dose-dependent effects of EGCG on cognitive functions and brain pathology of experimental models, this study aimed to examine the effects of EGCG administration with doses of 25, 50, 100 mg/kg on cognitive deficits and some relevant molecular factors in Alzheimer's model rats.

## 2 Materials and methods

### 2.1 Animals

The animal experimental protocols received permission from the Biomedical Ethics Committee of Mashhad University of Medical Sciences (Code: IR.MUMS.AEC.1401.029). All procedures in the animal experiments strictly followed the guidelines for the care and use of laboratory animals issued by the National Institutes of Health. Extensive efforts were made to alleviate the suffering and decrease the number of animals utilized in the present study.

### 2.2 Study design

**Fig. 1:**
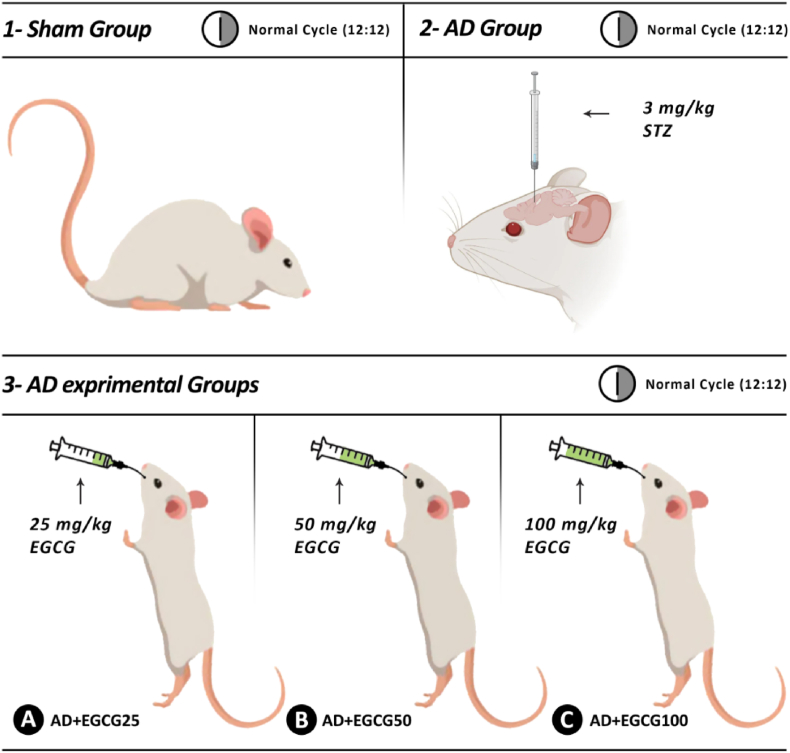
Grouping of rats based on Alzheimer's induction and EGCG administration.

Forty adult male Wistar rats, weighing between 180 and 200 g, were acquired from the Laboratory Animal Care Center at the Faculty of Medicine in Mashhad, Iran. Rats were maintained in the experimental animal facility under controlled conditions of 24 ± 2 °C, with a 12-h light/dark cycle, and were provided with unrestricted access to food and water. Following one week of adaptive feeding, rats were randomly assigned to five groups (n = 8): sham, AD, and AD + EGCG at three doses (Fig. 1).

### 2.3 AD model establishment

The animal was anesthetized by intraperitoneal injection of a mixture of ketamine (ketamine 80–100 kg/mg IP) and xylazine (10 kg/mg). After being placed in the stereotaxic machine, two holes were made on the surface of the skull with coordinates: Posterior mm 3.6: Ventral, 1.5 mm: Lateral, 0.8 mm was created in the region of the lateral ventricles of the brain, and then two number 26 needles were implanted as cannulas using dental cement. STZ was administered at a dosage of 3 mg/kg in 2 μL of saline via a Hamilton syringe into the lateral ventricles. Pain relievers were prescribed to alleviate discomfort, and antibiotics were administered to prevent infection. The injection rate was 0.5 μl/min. To prevent the return of the injected liquid, the needle head was maintained in position for 3 min. Following this, the needle was slowly removed after 3–5 min, and the rats were sterilized and sutured. The STZ injection was administered again two days later at the same dosage [12]. To ensure the induction of Alzheimer's, pilot tests were performed on the main groups before the experiment. The research steps are shown in Fig. 1.

### 2.4 EGCG administration

EGCG was purchased from Carbosynth Ltd, UK (CAS No: 989-51-5). A certain amount of EGCG powder was dissolved in a specific volume of saline and gavage was performed with doses of 25, 50, and 100 mg/kg in a volume of 4 ml once a day for 21 days (Fig. 1). These doses were chosen based on a previous study [13] and also to cover the range of doses from low to high applied in different studies.

### 2.5 Morris water maze test

The Morris water maze (MWM) test assessed the learning, memory processes, and spatial abilities of rats. The MWM system comprises a circular pool made of stainless steel, measuring 120 cm in diameter and 50 cm in height. The water temperature was regulated at 20 ± 1 °C. A camera linked to the display system was positioned at the top of the water maze to document the trajectory over time.

Behavioral testing was conducted over two consecutive days in this study. On the initial day, the spatial learning of the animals was assessed through three blocks conducted 30 min apart, with each block comprising four trials. In each trial, the animal was released into the water from one of four quadrants, randomly selected by the machine, with the animal's head oriented towards the maze wall. The animal had a maximum of 60 s to locate the hidden platform beneath the water's surface and rest on it, utilizing the surrounding spatial cues. If the animal did not locate the platform within 60 s, the researcher manually guided it to the platform. Upon placement on the platform, the animal rested for 30–35 s, followed by an additional 30–35 s of rest inside the cage and beneath the lamp. The following trials were conducted in a similar manner by releasing the animal from the alternate quadrants of the circle. In each block, the animal was released into the water from four distinct quadrants of the circle. The escape latency and the distance traveled to locate the hidden platform in these three blocks were assessed to evaluate the animals' spatial learning. Upon completion of the third block, the animals were thoroughly dried using a hair dryer and subsequently relocated to their cages. Twenty-four hours later, a probe test was conducted to assess the spatial memory of the animals. The test involved a single trial during which the hidden platform was absent from the maze. The animal was released into the water from the quarter opposite the target circle, allowing it to swim freely for 60 s. Consequently, the variables examined in this study included the duration of attendance, the distance traveled within the quadrant of the circle where the platform was previously situated (Quadrant Target), and the frequency of entries into this quadrant [14].

### 2.6 RNA isolation and quantitation

The TRIzol reagent kit was used for RNA extraction and purification. For this purpose, first, RNA is converted into cDNA (Complementary DNA) by reverse transcription; then, a real-time PCR reaction is performed on cDNA. After removing the DNA, the prepared RNA was used as a template in the reverse polymerase (RT) reaction. In this study, the expression of BDNF and APP genes was investigated. Primer sequences synthesized by Pishgaman Biotechnology Company (Tehran, Iran) are shown in Table 1.

**Table 1:** The primer sequences of RT-qPCR.

Gene	Primer sequences
**APP**	F: 5′-GCGGCAACAGGAACAACTTT-3′
	R: 5′-TGCCGTCGTGGGAAACAC-3′
**BDNF**	F: 5′-TTGTGTGGACCCTGAGTTCC-3′
	R: 5′-CAGCCTTCATGCAACCGAAG-3′
**GAPDH**	F: 5′-GATGGTGAAGGTCGGTGTGA-3′
	R: 5′-TGAACTTGCCGTGGGTAGAG-3′

Notes: RT-qPCR, reverse transcription-quantitative polymerase chain reaction; APP: Amyloid Beta Precursor Protein; BDNF: Brain-derived neurotrophic factor; GAPDH, glyceraldehyde-3-phosphate dehydrogenase.

### 2.7 Statistical analysis

SPSS software (version 16) was used for statistical calculations. Analysis of variance (ANOVA) and repeated measures were used to compare quantitative variables between independent or paired groups. If the ANOVA test was significant, appropriate post-hoc tests were used. If the tests' assumptions were unmet, equivalent non-parametric tests were used. A significance level of P < 0.05 was considered and the values are presented as means ± SEM.

## 3 Results

### 3.1 Elapsed time to find the target platform in learning phase of MWM

**Fig. 2:**
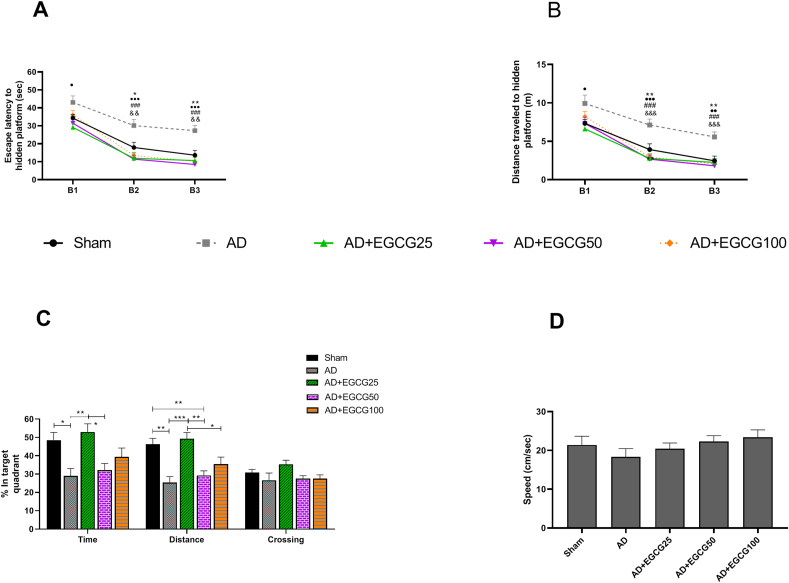
(A) Comparison of the time taken to reach the platform in the MWM between different experimental groups. Data are shown as mean ± standard error of the mean. (B) Comparison of the distance traveled to reach the platform in the Mauritius MWM between different experimental groups. (C) Comparison of the percentage of time and distance traveled and crossing the quadrant of the circle where the platform was located between different test groups in the probe test phase. (D) Speed comparison between different experimental groups. (Data are shown as mean ± SEM. In A and B: ∗P < 0.05, ∗∗P < 0.01 compared to sham group, and • P < 0.05, •• P < 0.01, … *p* < 0.001compared to AD + EGCG25, and ###P < 0.001 compared to AD + EGCG50, and && P < 0.01, &&& P < 0.001compared to AD + EGCG100.

The results indicated that STZ impaired rats' performance during three learning blocks and caused the AD group animals to have a longer delay in reaching the platform than the sham group and other groups (Fig 2A). Tukey's between-group comparison test showed that the performance of AD group animals disrupted in the first block compared to the AD + EGCG25 group (P < 0.05). AD group Also showed a disrupted performance in block 2 (P < 0.05, 0.001, 0.001, and 0.01) and block 3 (P < 0.01, 0.001, 0.001, and 0.01) in comparison with sham, AD + EGCG25, AD + EGCG50, and AD + EGCG100 respectively.

### 3.2 Distance traveled to find the target platform in learning phase of MWM

The comparison between groups indicates that the animals of the AD group traveled a greater distance to reach the platform than the Sham group and other groups (Fig. 2B). Tukey's between-group comparison test showed that the performance of AD group animals disrupted in the first block compared to the AD + EGCG25 group (P < 0.05). AD group Also showed a disrupted performance in block 2 (P < 0.01, 0.001, 0.001, and 0.001) and block 3 (*P* < 0.01, 0.01, 0.001, and 0.001) in comparison with sham, AD + EGCG25, AD + EGCG50, and AD + EGCG100 respectively.

### 3.3 Time, distance, and crossing over the target guardant in memory phase of MWM

The results of the comparison of spatial memory in the MWM are shown in Fig. 2C. The results showed that the percentage of time spent in the target quarter in the AD group was reduced compared to the sham group (P < 0.05), and the dose of 25 mg/kg of the active substance of green tea in the AD + EGCG25 group was able to significantly restore it to the level of sham group (P < 0.01). Also, the AD + EGCG25 group performed better than the AD + EGCG50 group (P < 0.05).

Regarding the distance traveled variable in the target quarter, the AD group showed a reduced performance compared to the sham group (P < 0.01). In contrast, the mentioned variable in the AD group treated with a dose of 50 kg/mg is still significantly lower compared to the sham group (P < 0.01). However, the dose of 25 kg/mg has been able to improve this variable compared to the AD group (P < 0.001), AD + EGCG50 group (P < 0.01), and the AD + EGCG100 group (P < 0.05). The change of crossing on the target quadrant did not show a significant change between the groups.

### 3.4 Speed in MWM test

Fig. 2D shows the average speed of the rats in the first block. As it is known, there was no significant difference between the groups.

### 3.5 mRNA expression of APP and BDNF in the hippocampus of rats

**Fig. 3:**
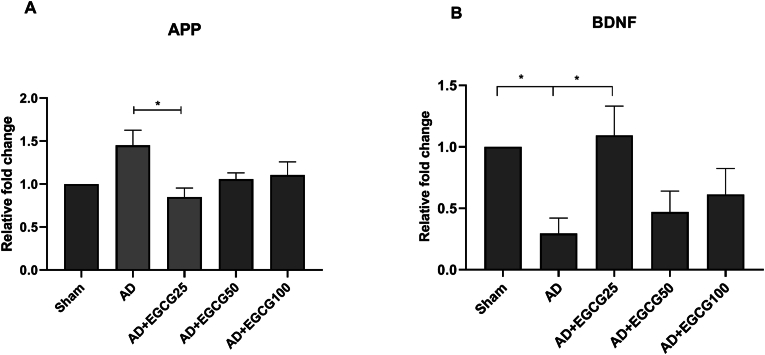
(A) Comparison of APP gene expression between different experimental groups. (B) Comparison of BDNF gene expression between different experimental groups. (Data are shown as mean ± SEM. ∗P < 0.05.

As shown in Fig. 3A, the expression of APP increased in the AD group compared to the sham group, but it didn't reach a significant level (*P* > 0.05). However, the dose of 25 mg/kg of the effective substance decreased the expression of this gene compared to the AD group (P < 0.05).

In Fig. 3B, the BDNF gene expression in the AD group decreased compared to the sham group (P < 0.05), and the dose of 25 mg/kg of the effective substance increased its level to that of the sham group (P < 0.01).

## 4 Discussion

The present study aimed to examine the effect of EGCG administration with doses of 25, 50, 100 mg/kg on cognitive deficits and some molecular memory factors in STZ induced Alzheimer rat model. The data showed that STZ disrupt the learning parameters of animals in MWM test and all doses of EGCG could improve the learning ability in acquisition phase. In the probe trial in which the retrieval of spatial memory performance in MWM task was measured, STZ also impaired the memory function and a dose of 25 mg/kg EGCG could significantly return it to the control level. The AD animals treated with dose 25 of EGCG had better memory performances compared with animals received dose 50 and 100 of EGCG. The swimming speed of the animals in the MWM did not show any significant difference.

Regarding the molecular markers of memory, we found that the dose 25 mg/kg of EGCG decreased the elevated expression of APP within the hippocampus of AD rats. BDNF gene expression in the AD group decreased and the dose of 25 mg/kg EGCG increased its level to that of the sham group.

Various substances and toxins can be used to induce AD in laboratory animals. STZ is one of the most commonly used compounds for experimentally AD induction [15]. STZ is a glucosamine-nitrosourea derived from the microbe Streptomyces chromogens. Intracerebroventricular (ICV) injection of STZ disrupts glucose and energy metabolism in the brain, which can be a central event in the initiation of neurodegeneration seen in AD [16]. ICV administration of low doses of STZ, by changing insulin receptor tyrosine kinase activity and disrupting insulin receptor signaling, causes insulin resistance in the brain, followed by a decrease in glucose and energy metabolism [17]. STZ is usually diluted in saline, citrate buffer, or artificial cerebrospinal fluid. It is administered in one or more injections in the range of 1–3 mg/kg, unilaterally or bilaterally in the lateral ventricles of the brain, with acute-rapid memory impairment, followed by a partial recovery and then a chronic-slow memory disorder is identified [17]. Accordingly, in our study, STZ caused significant memory impairments.

A 2012 study by Tao Xie and colleagues investigated the effects of EGCG on seizures, oxidative stress, and cognitive deficits in kindled model induced by PTZ in male rats. EGCG doses of 25 mg/kg and 50 mg/kg were administered along with PTZ injections, showing dose-dependent suppression of kindling progression and improved cognitive deficits and oxidative stress. EGCG with dose 50 mg/kg was more effective in suppressing the progression of kindling and enhancing memory deficits in kindled animals [18].

Young Kyoung Lee and colleagues' study in 2009 examined the preventive effects of EGCG on memory impairment induced by lipopolysaccharide (LPS). Oral treatment with EGCG (1.5 and 3 mg/kg) for three weeks showed dose-dependent prevention of Aβ increase by inhibiting β- and γ-secretase activities induced by LPS. EGCG also prevented neuronal cell death, expression of inflammatory proteins, inducible nitric oxide synthase, and cyclooxygenase-2. The study demonstrated that the 3 mg/kg dose significantly improved cognitive deficits and pathological changes induced by ICV injection of LPS in Alzheimer's-like rats [19]. Both studies confirmed the neuroprotective properties of EGCG, although Lee et al. highlighted the dose-dependent effects of EGCG.

Jie et al. investigated the effects of EGCG on high-frequency stimulation-induced long-term potentiation (LTP) in the Schaffer collateral-CA1 synapse with or without ischemic brain injury caused by middle cerebral artery occlusion. The study showed that EGCG dose-dependently facilitated LTP in healthy rats, although relatively high doses of EGCG inhibited the induction of LTP in healthy rats [13].

Xiang et al., in 2015 examined the effects of EGCG at high and low doses (5 and 15 mg/kg) on β-amyloid accumulation and cognitive decline in rats. They reported that both high and low doses of EGCG reduced β-amyloid accumulation and MDA levels and alleviated cognitive decline in rats [20].

In another study, a protective effect of EGCG against cerebral ischemia-induced brain damage in gerbils was reported. EGCG (25 or 50 mg/kg) 30 min before and immediately after ischemia reduced excitotoxin-induced malondialdehyde (MDA) production and neuronal damages. In the in vivo experiments of this study, low dose of EGCG failed to demonstrate neuroprotective effects [21].

Although these studies utilized the different models, they corroborated the positive effects of green tea ingredient as a neuroprotective agent. In our study, high doses showed a slight trend toward the improvement of memory and molecular changes in AD rats, but low dose (25 mg/kg) only showed significant effectiveness. Ding et al. reported that the enhancing effect of EGCG is in a dose-dependent manner.

The protective effects of green tea or EGCG against the neurotoxicity and neurological damages caused by beta-amyloid have been partially studied [22,23]. However, the robust effect and molecular mechanisms of EGCG have not been well understood. The results obtained from our study showed that EGCG at a dose of 25 mg/kg of body weight can improve the memory function of AD model rats by reducing β-amyloid precursor protein (APP) and increasing brain-derived neurotrophic factor (BDNF).

BDNF is a neurotrophin that regulates neuron survival, differentiation, and plasticity by activating tyrosine kinase B receptor (TrkB) and neurotrophin receptor 75P (NTR 75P). Following the reduction of BDNF, it leads to impairment in spatial memory, while overexpression of TrkB enhances memory, and BDNF signaling through TrkB causes long-term strengthening of hippocampal synapses [24]. BDNF plays a vital role in promoting the recovery of the nervous system by neurogenesis, regulating sprouting, synaptogenesis, and eliminating the unwanted synapses [25].

The β-amyloid precursor protein (APP) is broken down to form β-amyloid (Aβ), which accumulates in the brain in AD. Increased APP gene sequence or expression may affect Aβ levels and disease risk. The limit of typical APP sequence leads to an increase in total Aβ and ultimately to the rise in Aβ accumulation, which leads to dementia and neuropathology of AD [26].

As the lowest dose of EGCG in our findings could significantly affect both the memory and molecular changes seen in AD animals, it is reasonable to postulate that the memory deficits observed in AD rats may be in part due to the increased APP and decreased BDNF gene expression within the hippocampus of AD animals. More studies are needed to confirm these observations.

Some studies have reported that the higher doses of EGCG exhibit more neuroprotective potentials than lower doses [18,19,21]. However, our study showed the opposite findings so that the EGCG with lowest dose, ie 25 mg/kg, seems to have more improving effect compared to 50 and 100 mg/kg. The reported variation in findings can be attributed to the different behavioral protocols and experimental animals used, duration of EGCG consumption, and variation of how the authors analyze and interpret their observations. Further investigations are needed to conclude with certainty about the most efficient therapeutic dose.

## 5 Conclusion

The results of the present study showed that the oral administration of active ingredient of green tea, EGCG, at a dose of 25 mg/kg has a significant improving effect on cognitive deficits in Alzheimer – like model rats. These effects could be in part due to the parallel changes of hippocampal neurogenesis markers observed in the same animal group. These findings could provide a beneficial insight for developing novel, safe, and efficient natural compounds for preventing or alleviation AD symptoms in humans. However, more studies are needed to determine the benefits of different doses of EGCG and the plausible mechanisms corresponding to these beneficial effects. Especially for neurogenesis analysis, using more specific neuronal markers such as NeuN or BrdU could increase the confidence of our conclusions.

## CRediT authorship contribution statement

**Farnaz Ghayour Babaei:** Writing – original draft. **Ehsan Saburi:** Conceptualization. **Fatemeh Forouzanfar:** Formal analysis. **Mohadese Asgari:** Writing – original draft. **Zakieh Keshavarzi:** Writing – review & editing. **Vahid Hajali:** Supervision.

## Data availability

Data will be made available on request.

## Founding information

This study was supported by a grant (IR.MUMS.AEC.1401.029) from the research assistance of 10.13039/501100004748Mashhad University of Medical Sciences, Mashhad, Iran. The authors did not receive support from any organization except MUMS.

## Declaration of competing interest

The authors declare that they have no known competing financial interests or personal relationships that could have appeared to influence the work reported in this paper.
